# Dos and don'ts in large animal models of aortic insufficiency

**DOI:** 10.3389/fvets.2022.949410

**Published:** 2022-09-02

**Authors:** Miriam Weisskopf, Lukas Glaus, Nina E. Trimmel, Melanie M. Hierweger, Andrea S. Leuthardt, Marian Kukucka, Thorald Stolte, Christian T. Stoeck, Volkmar Falk, Maximilian Y. Emmert, Markus Kofler, Nikola Cesarovic

**Affiliations:** ^1^Center for Surgical Research, University Hospital Zurich, University of Zurich, Zurich, Switzerland; ^2^Department of Health Sciences and Technology, Swiss Federal Institute of Technology, Zurich, Switzerland; ^3^Department of Cardiothoracic and Vascular Surgery, German Heart Center Berlin, Berlin, Germany; ^4^Institute for Biomedical Engineering, University and ETH Zurich, Zurich, Switzerland; ^5^Department of Cardiovascular Surgery, Charité-Universitätsmedizin Berlin, Berlin, Germany

**Keywords:** large animal model, aortic valve, paravalvular leakage, aortic insufficiency (AI), Minimally invasive, PV loop, MRI, pig

## Abstract

Aortic insufficiency caused by paravalvular leakage (PVL) is one of the most feared complications following transcatheter aortic valve replacement (TAVI) in patients. Domestic pigs (*Sus scrofa domestica*) are a popular large animal model to study such conditions and develop novel diagnostic and therapeutic techniques. However, the models based on prosthetic valve implantation are time intensive, costly, and often hamper further hemodynamic measurements such as PV loop and 4D MRI flow by causing implantation-related wall motion abnormalities and degradation of MR image quality. This study describes in detail, the establishment of a minimally invasive porcine model suitable to study the effects of mild-to-moderate “paravalvular“ aortic regurgitation on left ventricular (LV) performance and blood flow patterns, particularly under the influence of altered afterload, preload, inotropic state, and heart rate. Six domestic pigs (Swiss large white, female, 60–70 kg of body weight) were used to establish this model. The defects on the hinge point of aortic leaflets and annulus were created percutaneously by the pierce-and-dilate technique either in the right coronary cusp (RCC) or in the non-coronary cusp (NCC). The hemodynamic changes as well as LV performance were recorded by PV loop measurements, while blood flow patterns were assessed by 4D MRI. LV performance was additionally challenged by pharmaceutically altering cardiac inotropy, chronotropy, and afterload. The presented work aims to elaborate the dos and don'ts in porcine models of aortic insufficiency and intends to steepen the learning curve for researchers planning to use this or similar models by giving valuable insights ranging from animal selection to vascular access choices, placement of PV Loop catheter, improvement of PV loop data acquisition and post-processing and finally the induction of paravalvular regurgitation of the aortic valve by a standardized and reproducible balloon induced defect in a precisely targeted region of the aortic valve.

## Introduction

### Background

New onset of aortic regurgitation is considered one of the major complications following aortic valve replacement interventions. Regurgitant flow between the prosthetic valve and native aortic tissue is termed paravalvular leakage (PVL), and it is often caused by malapposition of the two structures and consequent insufficient sealing. Even with new generation transcatheter heart valves, implanted in patients with favorable device landing zone (tricuspid aortic valve, no severe calcification of the left ventricular outflow tract) the rate of at least mild PVL is still around 30% (1) and increases to almost 60% for patients with less favorable landing zones. In patients, most PVLs are considered hemodynamically insignificant; however, contemporary literature suggests that even mild PVL is associated with worse postoperative survival (2). Experimentally it could be shown that already a mild-to-moderate PVL alters intraventricular blood flow patterns by disturbing physiologic vortex formation thereby causing a potential increase in dissipation of left ventricular energy (3). All circumstances altering the cardiac output need to be interpreted in the light of the ongoing trend to use transcatheter heart valves in younger and physically more active patients. High fidelity animal models with mild-to-moderate PVL are an unmet need to provide evidence for the significance of such fluid dynamic alteration in relation to reduced cardiac function.

Rosa et al. previously described a porcine model of PVL created by replacing the native aortic valve with a lab-fabricated one, while intentionally leaving space for regurgitant flow between the valve and the aortic wall (4). Although the model appears to offer opportunities to develop and test therapeutic approaches to reduce or eliminate regurgitant flow, the model is not only difficult to reproduce but also hampers the assessment of blood flow characteristics in detail with 4D flow MRI, as image quality is degraded, dependent on the stent type and stent orientation relative to the magnetic field (5). To overcome this limitation, we developed a large animal model of mild PVL by transcatheter piercing of the aortic valve at the hinge point of the annulus and leaflet (3). Following the selection of appropriately sized animals free from pathogens that could influence cardiac health, aortic valve defects are created in a percutaneous, transcatheter fashion using the pierce-and-dilate technique. In detail, under fluoroscopic and echocardiographic guidance, a steerable sheath is navigated to either the non-coronary (NCC) or right-coronary cusp (RCC) of the aortic valve. Once in position, a stiff coronary guide wire is used to pierce the leaflet-annulus hinge point. A coronary balloon is then passed over the wire and inflated to produce a standardized defect causing aortic regurgitation (i.e., PVL). For a successful assessment of the effects on left ventricular (LV) performance, it is paramount to establish a stable position of the PV loop catheter resistant to the manipulations during PVL creation, enabling direct comparison between the healthy state and immediate hemodynamic effects of PVL. Moreover, drugs can be used to alter the hemodynamic parameters, such as cardiac afterload (i.e., aortic pressure and resistance), heart rate, and contractility, known to influence the severity and negative effects of aortic regurgitation. The animal model thereby simulates also hypertensive and/or tachycardiac patients suffering from aortic regurgitation, potentially leading to a better understanding of underlying processes responsible for adverse cardiac remodeling in this population.

Patients suffering from severe PVL display clear symptoms and can benefit from the guideline directed therapy. However, for those patients displaying only mild-to-moderate PVL the situation is less clear. This population displays a wide variety of symptoms and a therapeutic spectrum ranging from disease monitoring to invasive interventions. Moreover, currently, the influence of the PVL jet origin and its effects on left ventricular flow and function are largely left unconsidered. Hence, the main purpose of this model is to provide a reproducible, cost-effective, and ethically justifiable translational large animal platform to investigate the fluid dynamic and energetic effects of mild-to-moderate aortic PVL originating from the areas of the aortic valve clinically associated with the presentation of PVL namely the right- or the non-coronary cusp.

The presented work aims to elaborate on the dos and don'ts when establishing an aortic insufficiency/PVL model for hemodynamic studies. Benefitting from each other's learning curves will reduce redundancy in animal research and improve data quality in accordance with the 3R principles established worldwide as the ethical approach to regulating the use of animals for scientific purposes (6).

### Selection of appropriately sized animals free of cardiac pathogens is a key prerequisite

The use of the domestic pig (*Sus Scrofa Domestica*) in preclinical studies plays an important role in the evaluation of efficacy and safety of new cardiac medical devices before their use in human clinical trials (7–9). However, when planning and executing porcine trials, a variety of aspects should be taken into consideration. The health status of domestic pigs varies greatly depending on their origin; thus, a rigorous pre-screening for diseases affecting cardiac health and function should be performed. A multitude of infectious diseases and nutritional deficiencies can alter cardiovascular health in commercial pigs. Among the most important infectious agents are thereby the *Porcine Circovirus type 2 (PCV2)*, which has previously been associated with heart failure in young pigs due to acute necrotizing or chronic fibrosing myocarditis, and chronic vasculitis in multiple organs (10). The *Encephalomyocarditis Virus* (EMCV) causing acute myocarditis and sudden death in preweaned pigs and reproductive failure in sows (11) has become of recent concern in the context of xenotransplantation. Although it is described as asymptomatic in older pigs, the virus appears to persist in the myocardium over a prolonged period (11). *Hemophilus parasuis*, the cause of Glasser's disease, commonly causes serofibrinous pleuritis, pericarditis, peritonitis, and arthritis (12). Porcine endocarditis is another well-known disease entity, with *Streptococcus suis* and *Erysipelothrix rhusiopathiae* being the dominant bacteria isolated from infected heart valves (13). Mulberry heart disease in pigs is characterized by lesions of acute hemorrhagic myocarditis and myocardial necrosis and has been associated with vitamin E/selene deficiency (14), recent studies further suspect viral association in the disease (15).

Anatomical differences between pigs and humans are a further aspect to consider when using this animal model. Most differences thereby arise from pigs being quadruped, hence having a narrower thorax in latero-lateral rather than in dorso-ventral orientation, unlike humans that feature a dorso-ventrally “compressed” chest (16). Consequently, the major cardiac axis of the porcine heart is tipped ventro-caudally and forms a steeper angle. Nonetheless, a vast majority of cardiac dimensions in pigs appear to correlate well with the average adult human (16), though left atrial volume and dimensions can be smaller in pigs than equivalent mitral annulus size seen in humans (17). Furthermore, the relative cardiac mass in certain breeds of pigs appears to decrease significantly from 5 gr/kg BW to just 2.3–2.9 gr/kg BW, as maturity is reached (18). As pre-experimental selection of pigs is commonly based on body weight, this nonlinear correlation between body mass and cardiac size needs to be considered. In general, in trans-catheter aortic valve implantations (TAVI) in pigs, complications are often associated with over- or undersizing of the valve and are comparable to those seen in humans, namely, coronary occlusion, rupture of the aortic root or annulus post-implantation, PVL or transcatheter valve migration (19, 20). Cardiac valve, in particular, aortic valve sizing in preclinical trials requires a different approach than typically used in clinics. Compared to the diseased calcified annulus of patients, the annulus of a healthy pig is more dynamic during the cardiac cycle demanding appropriate oversizing to avoid migration and stability issues hampering sufficient sealing between the prosthetic valve and the aortic wall (21).

### Combination of fluoroscopy and ultrasound imaging is necessary to guide PVL creation and clinically assess its position and severity

Fluoroscopy was used to image the position of the steerable sheath, however, as it does not allow visualization of cardiac soft-tissue structures, which is needed to provide adequate visualization of the targeted cardiac anatomy necessary for the successful PVL creation, fluoroscopy is best complemented with ultrasound. While transthoracic echocardiography (TTE) in pigs is technically challenging by the previously mentioned keel-shaped thorax and by narrowly spaced ribs (22), a three-chamber view, showing a long axis of the left ventricle and the aortic valve can be obtained by placing a trans-esophageal echo (TEE) probe in the mid-esophageal position in an ante-flexed and latero-flexed position and plane rotating the transducer between 90 and 130° (23, 24), adjusting rotation as needed to account for individual anatomy of the animal. To obtain a simultaneous short axis view of the left ventricular outflow tract and the aortic valve, an intracardiac echocardiography (ICE) probe can be introduced through the femoral vein and placed at a low RA location in a postero-flexed position followed by counterclockwise rotation by 60–100° (25). Combining these imaging modalities allows for a precise and reproducible aortic valvular leaflet piercing.

### 4D MRI is best complemented by PV loop analysis to assess the effects of PVL on LV function

For a comprehensive evaluation of PVL on left ventricular hemodynamics, 4D MRI flow data is best complemented with left ventricular PV loop analysis, which is considered to be the gold standard for assessing cardiac function during each cardiac cycle (26). PV loops offer invaluable insights into the pathophysiology of PVL in the porcine model. However, PV loop analysis not only requires knowledge of the pig's anatomical and physiological particularities but comprehensive analytical skills for adequate data processing. Hence, comprehensive preclinical studies highly benefit from interdisciplinary collaborations of veterinarians, physicians, and engineers, to ensure animal welfare, translatability of the study, and reproducibility by high-quality data.

## Materials and equipment

Animals

- Large white pigs, female, 60–70-kg body weight

Drugs

- **Ketamine** (Ketasol^®^-100 ad us.vet.; Dr. E. Graeub AG, Berne, Switzerland; 15-mg/kg body weight).
- **Azaperon** (Stresnil^®^ ad us.vet.; Elanco Tiergesundheit AG, Basel, Switzerland; 2-mg/kg body weight).
- **Atropin 1%** (Atropinsulfat KA vet 0.1%; Kantonsapotheke, Switzerland; 0.05-mg/kg body weight).
- **Vitamin A Eye Ointment** (Vitamin A Blache Augensalbe, Bausch & Lomb Swiss AG, Zug, Switzerland).
- **Propofol** (Propofol ^®^ Lipuro 1%, B. Braun Medical AG; Sempach, Switzerland; 1–2 mg/kg body weight (bolus), 3–5 mg/kg/h continuous infusion).
- **Isoflurane** (Attane™ Isoflurane ad.us.vet., Piramal Enterpr. India, Lyssach, Switzerland, 1–2%).
- **Buprenorphine** (Temgesic^®^ Indivior Schweiz AG, Baar, Switzerland; 0.01 mg/kg).
- **Amiodarone** (Cordarone^®^, Sanofi-Aventis (Suisse) SA, Vernier, Switzerland, 150 mg/100 ml 5% glucose, slow drip to effect).
- **Ringer's solution** (Ringerfundin^®^, B. Braun Medical AG, Sempach, Switzerland, 5 ml/kg/h).
- **Sodium-Heparin** (Na-Heparin, B. Braun Medical AG, Sempach, Switzerland).
- **Dobutamine** (Dobutrex^®^, Teva Pharma AG, Basel, Switzerland, 2.5 mg/ml, continuous infusion to effect).
- **Phenylephrine** (Neo-Synephrine HCl, Ospedialia AG, Hünenberg, Switzerland, 0.1 mg/ml, continuous infusion to effect).
- **Hypertonic Saline** (Natrium Chloratum Sintetica 25%, Sintetica S.A., Mendrisio, Switzerland,0.25 ml/kg 15% hypertonic NaCl).

### Materials

- **Intravenous catheter** (BD Venflon ™ Pro Safety, Becton Dickinson Infusion Therapy, Helsingborg, Sweden, 18G).
- **Endotracheal tube** (Bivona^®^, ID: 9 mm, OD: 12.4 mm, 37FR, Length: 56 cm, Balloon: 5 cm).
- **Urinary balloon catheter** (Rüsch^®^, Ch 12, Teleflex Medical GmbH, Belp, Switzerland).
- **Vascular access sheath** (Avanti^®^+ Introducer,6–10 F, Cordis^®^, Miami Lakes FL, USA).
- **Intra-cardiac echocardiography (ICE) probe** (ViewFlex™ Xtra ICE Catheter, St.Jude Medical, Minnesota, USA).
- **Transesophageal echo probe** (X7-2T TEE Transducer, Philips, Amsterdam, The Netherlands).
- **Bidirectional steerable guiding sheath** (Agilis™ EPI Steerable Sheath, 8.5F, St. Jude Medical, Minnetonka, MN, USA, Or DESTINO™ Reach 8.5F ID, usable length 77cm, curve 17mm, Oscor^®^, Florida, USA).
- **PV loop catheter** (Ventri-Cath Catheter; 5F, 12E, 10 mm, DField, Pigtail, 122 cm; ADInstruments Limited, Oxford, United Kingdom).
- **Pig-tail catheter** (Infiniti 5F PIG 145.038 125 cm, Cordis Corporation, Miami Lakes, USA).
- **J-tip Guide Wire** (Rosen, Heavy-Duty Corewire J-tip, 0.035”, 180 cm, Cordis^®^, Miami Lakes FL, USA).
- **Extra-Stiff guide wire** (Lunderquist^®^, 0.035”260 cm, Cook Medical, Bjaeverskov, Denmark).
- **Coronary guide wire** (IRON MAN Guide Wire, 0.014″ 190 cm, Abbott Vascular, Santa Clara CA, USA).
- **PTCA balloon** (NC Emerge MONORAIL™ PTCA Dilation Balloon 5 × 12 mm, Boston Scientific Corporation, Marlborough, MA, USA).

### Equipment

- **Anesthesia machine** (Dräger Fabius GS, Dräger Medical, Lübeck, Germany).
- **Anesthetic monitoring** (Dräger Infinity, Dräger Medical, Lübeck, Germany).
- **Fluoroscopy C-Arm** (Allura Xper FD20, Philips Healthcare, Horgen, Switzerland).
- **Ultrasound machine** (TEE) (iE33, Philips Healthcare, Horgen, Switzerland).
- **Ultrasound machine** (ICE) (CX50, Philips Healthcare, Horgen, Switzerland).
- **DSI Ponemah System** (DataScience International, St. Paul, Minnesota, USA).
- Millar Mikro-Tip^®^ Pressure Volume System (MPVS) Ultra Foundation Systems (AD Instruments, Oxford, United Kingdom).
- **Powerlab 16/35 Acquisition Unit** (AD Instruments, Oxford, United Kingdom).
- **Clinical 3T MR Scanner** (Ingenia, Philips Healthcare, Best, the Netherlands).

## Methods

### Animal acquisition, health check, and anesthesia

Six domestic pigs (*n* = 6; Swiss large white, intact females, 60–70 kg of body weight) were included in this study approved by the local Committee for Experimental Animal Research (Cantonal Veterinary Office Zurich, Switzerland) under the License numbers ZH213/2019. Animal housing and all experimental procedures were in accordance with the Swiss animal welfare protection law and conformed to the European Directive 2010/63/EU of the European Parliament and the Council on the Protection of Animals used for Scientific Purposes, and to the Guide for the Care and Use of Laboratory Animals. The pigs used in this study were all screened under the national surveillance program for Classical and African Swine fever, Foot and Mouth disease, Aujeszky's disease, Porcine reproductive and respiratory syndrome (PRRS), and Swine vesicular disease. Piglets are vaccinated against *H. parasuis* and *Porcine Circovirus* at the age of 3 weeks and 6 weeks. Sows are vaccinated at the age of 5–7 months and repeatedly prior to giving birth against Parvovirus and *E. rhusiopathiae*. Additionally, sows are vaccinated against *Escherichia coli* at 5 and 3 weeks prior to giving birth. Upon arrival, all pigs should be clinically assessed by observation (general behavior, posture and gait, visible injuries, color of the skin, breathing pattern, appetite, defecation, and urination) and if further indicated by physical examination.

Cardiopulmonary auscultation in the awake pig is not feasible as restraining the animal is stressful and will cause loud vocalization. The pigs used in this study were premedicated with an intramuscular injection of ketamine (Ketasol^®^-100 ad us.vet.; Dr. E. Graeub AG, Berne, Switzerland; 15 mg/kg body weight), azaperone (Stresnil^®^ ad us.vet.; Elanco Tiergesundheit AG, Basel, Switzerland; 2 mg/kg body weight), and atropine (Atropinsulfat KA vet 0.1%; Kantonsapotheke, Switzerland; 0.05 mg/kg body weight). Anesthesia was induced by an intravenous administration of propofol (Propofol ^®^ Lipuro 1%, B. Braun Medical AG; Sempach, Switzerland; 1–2 mg/kg body weight) to achieve relaxation and swallow-reflex diminishment sufficient for intubation. Anesthesia was then maintained with Isoflurane (Attane™ Isoflurane ad.us.vet., Piramal Enterpr. India; Lyssach, Switzerland, 1.5–3 vol%) in combination with 50% oxygen under spontaneous respiration, and in combination with a constant rate infusion of propofol (Propofol ^®^ Lipuro 1%, B. Braun Medical AG; Sempach, 5–10 mg/kg/h). For the pain medication, buprenorphine (Temgesic^®^ Indivior Schweiz AG, Baar, Switzerland; 0.01 mg/kg) was administered. All pigs received amiodarone (Cordarone, Sanofi-Aventis (Suisse) SA, Vernier, Switzerland, 150 mg/100 ml 5% Glucose) as an antiarrhythmic agent, already prior to instrumentalization. Ringer's solution (Ringerfundin^®^, B. Braun Medical AG, Sempach, Switzerland, 5 ml/kg BW/h) was infused throughout the experiment. A blood gas analysis was performed regularly and urinary output was monitored to be between 0.5 and 1 ml/kg BW/h and fluid substitution adjusted accordingly.

### Animal instrumentalization

Ultrasound-guided percutaneous placement of a femoral arterial introducer sheath (Avanti^®^+ Introducer, 6F, Cordis^®^, Miami Lakes FL, USA), a femoral venous introducer sheath (Avanti^®^+ Introducer, 10F, Cordis^®^, Miami Lakes FL, USA), and a left-sided carotid artery introducer sheath (Avanti^®^+ Introducer,6F, Cordis^®^, Miami Lakes FL, USA) was performed using the Seldinger method.

### PV loop preparation, placement, and data optimization

#### Bench preparation of the catheter

Prior to the experiment, the PV loop catheter and the rho calibration cuvette were placed into warm water (37°C) for 30 min. Placing the PV loop catheter in a long vascular access sheath thereby prevents misshaping of the catheter prior to use. During this period a 15% hypertonic saline solution for parallel conductance correction can be prepared, based on the recommendation of AD Instruments. By using a 15% hypertonic solution, a good calibration is achieved without adverse effects on the animal.

After the required immersion, a small amount of arterial blood (<1 ml) was drawn to measure resistivity, conductivity, and blood temperature with the rho cuvette. The final measurement should be taken when the temperature matches the expected core temperature of the animal. Major changes in blood composition or body temperature may warrant repeated intraprocedural rho cuvette measurements. The pressure sensor of the PV loop catheter is zeroed at body temperature by submerging the tip just below the surface of a physiological saline solution.

#### Catheter placement and *in-situ* calibration

**Figure 1:**
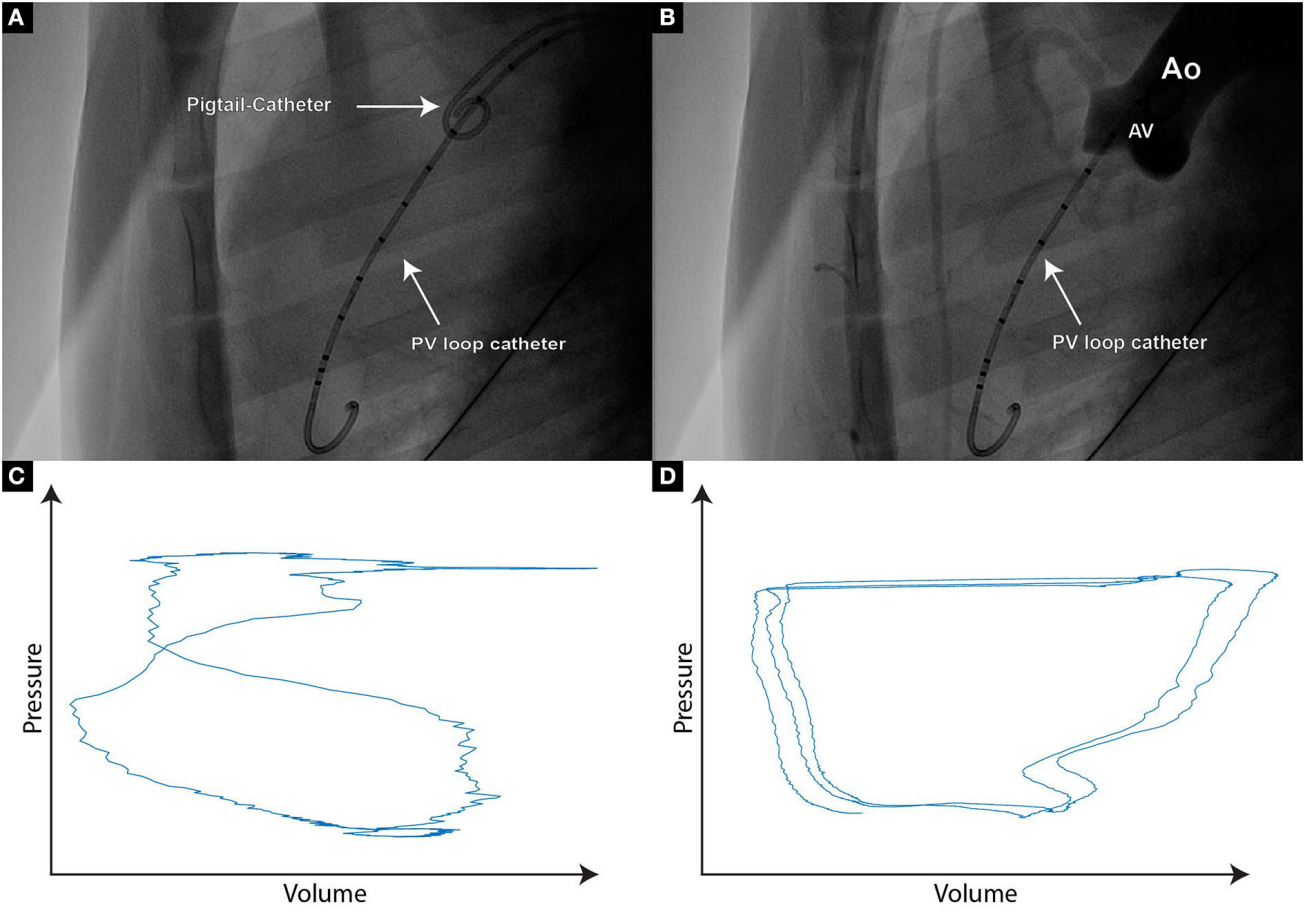
**(A)** Fluoroscopic image of a PigTail catheter which is positioned in the aortic valve cusp, hence enabling the assessment of PV loop catheter segments that are reliably positioned within the left ventricle; **(B)** If uncertainty regarding the PV loop segments within the left ventricle persists, contrast agent can be delivered through the PigTail catheter, hence providing an aortographic image clearly demonstrating the separation between the aorta and the left ventricle; **(C)** Typical “figure of 8” PV signal originating from the segment placed at the level of the aortic valve or above. Such segments should not be taken into further analysis; **(D)** Paradox composite PV loop curves demonstrating volume increase during systole and volume decrease during diastole. If such curves occur, re-positioning of the PV loop catheter needs to be considered.

Stonko et al. (27) previously described the optimal access point for the PV Loop catheter advancement in pigs to be through the right brachial artery, or either of the carotid arteries, with each of these access routes, the catheter can be naturally steered into the ascending aortic arch. However, as the carotid artery was used in our setting to introduce the steerable sheath for aortic leaflet piercing, we used the left femoral artery to insert the PV loop catheter. The lack of a guide wire lumen has shown to make PV loop catheter placement more challenging, as it needs to be navigated over the aortic arch to cross the aortic valve. To facilitate PV loop catheter steering over the arch and through the aortic valve a pigtail catheter can be placed in the aortic sinus to improve orientation during fluoroscopy. After positioning the PV loop catheter in the left ventricle, it needs to be ensured that there is no saturation of the segmental volume readings, which would present itself as maximal, horizontal lines and would require an increase of the volume gain in the MPVS software. In order to generate meaningful cumulative PV loops, the relevant intraventricular volume segments need to be determined. The pigtail catheter in the aortic sinus thereby helps visualize the level of the aortic valve, hence the number of PV loop catheter segments below the aortic valve can be easily determined in fluoroscopy (Figures 1A,B). Within LabChart, non-relevant segments can be recognized as crossing loops with the shape of the number 8 (Figure 1C).

By launching the large animal PV Loop workflow within LabChart, a 2-point calibration for pressure and volume can be performed by setting fixed voltages within the Millar MPVS Ultra software. The minimal and maximal volumes can be calculated by multiplying the number of segments with the minimal and maximal segment volume seen within MPVS Ultra. If the resulting cumulative PV loop of all segments shows skewed shapes like non-vertical isovolumetric contractions or relaxations, the positioning of the PV loop catheter is not ideal and should be adjusted (Figure 1D), ensuring that the catheter is in a steady position within the ventricle, without interfering with the chordae or the papillary muscles. Strong movements of the PV loop catheter might also indicate entanglement and thus require repositioning as well. A minor oscillation between the two end-diastolic volumes can be observed due to respiration and is generally accepted. Due to the constant cardiac contractions, the position of the PV loop catheter can change over time. Such position changes can cause changes in the measured parameters (particularly in LV volume) and make comparisons over time quite challenging. Hence, the catheter should be secured in place. We suggest that the fixation of the PV loop catheter is ensured with a generic soldering clamp clip that is placed in close proximity to the arterial access point.

The removal of parallel conductance volume originating from the myocardium can be achieved by rapid intravenous injection of the hypertonic saline solution according to the large animal PV workflow. To eliminate artifacts induced by respiration, mechanical ventilation is stopped for the duration of approximately five loops prior to injection and held for an additional five loops after returning to baseline intraventricular volume. High R^2^ values should be obtained with little variance in function of chosen loops, in order to obtain a robust parallel volume.

As a last calibration step, the measurements of the PV loop catheter are corrected for electrical field inhomogeneity by applying the alpha-correction, where the measured stroke volume of the PV loop catheter is compared with another type of measurement, such as echocardiography or MRI. In our case, we relied on TEE measurements for a simultaneous comparison. The integrity of the valves and cardiac movements are also verified by a TEE analysis.

### Baseline measurements

In order to measure congruent loops without respiratory artifacts, 20 PV loops are recorded without respiration, typically in the end-inspiratory position, allowing the plotting of the central 10 loops. For the derivation of hemodynamic parameters, such as stroke work, stroke volume, or cardiac output, we recommend acquiring loops over 1 min under mechanical ventilation, thus allowing averaging over a large number of loops.

### Interventions

#### Step 1: Placement of ICE and TEE probe

An intracardiac echocardiography (ICE) probe (ViewFlex™ Xtra ICE Catheter, St.Jude Medical, Minnesota, USA) was placed through the femoral venous access sheath, images were acquired on a Philips CX50 ultrasound machine (Philips, Amsterdam, the Netherlands). Furthermore, a transesophageal echo probe (X7-2T TEE Transducer, Philips, Amsterdam, the Netherlands) was inserted, and images were acquired on the Philips i33 ultrasound machine (Philips, Amsterdam, the Netherlands).

#### Step 2: Placement of steerable sheath

A bidirectional steerable guiding sheath (Agilis™ EPI Steerable Sheath, 8.5F, St. Jude Medical, Minnetonka, MN, USA, Or DESTINO™ Reach 8.5F ID, usable length 77 cm, curve 17 mm, Oscor^®^, Florida, USA) was introduced over an extra-stiff guide wire (Lunderquist^®^, 0.035″/260 cm, Cook Medical, Bjaeverskov, Denmark) through the left carotid artery.

The aortic arch of pigs differs in anatomy when compared to humans. There are two branch arteries arising from the aortic arch, first, the brachiocephalic trunk, which involves both common carotid arteries as well as the right subclavian artery, and second, the left subclavian artery (28). Although either common carotid artery arises from the brachiocephalic trunk, introducing the steerable sheath through the left carotid artery appears to be favorable in targeting the NCC or RCC of the aortic valve.

**Figure 2:**
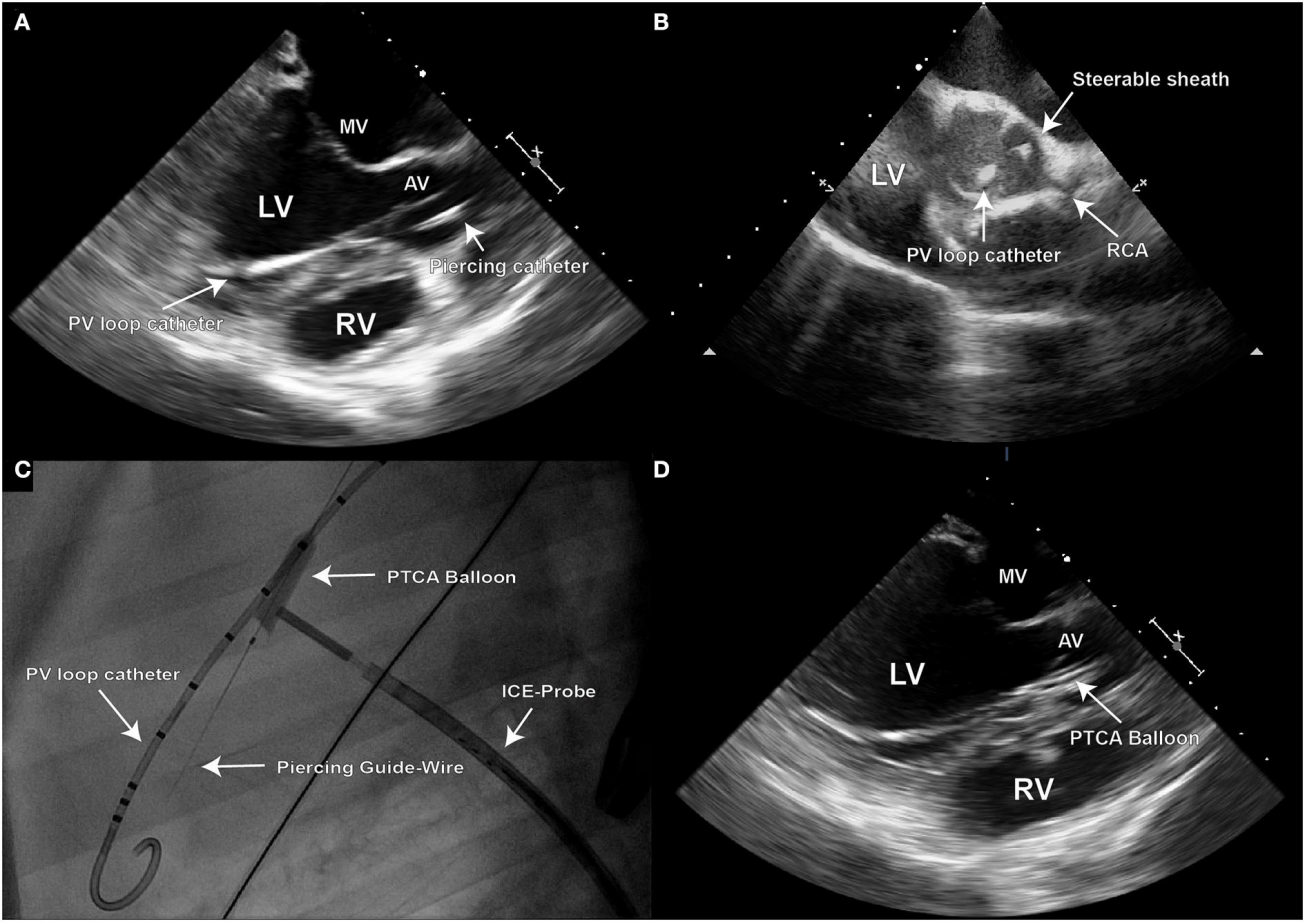
Due to the 2D character of echocardiographic imaging, two distinct perpendicular projections (long and short axis) are necessary for precise procedural navigation; **(A)** Transesophageal (TEE) long axis echo image demonstrating the final position of the steerable sheath on target for creation of the defect at the leaflet hinge point in the right coronary cusp (RCC) of the aortic valve; **(B)** Once such position is reached, intra-cardiac echo should be used to confirm the position in the short axis image; **(C)** Fluoroscopy is used to visualize the piercing guide wire during its positioning, and for control of the PTCA balloon inflation; **(D)** Inflated PTCA balloon used for defect dilation can be also visualized in TEE long axis view.

Positioning of the sheath was verified with TEE and ICE, as well as fluoroscopically (Figures 2A–C).

#### Step 3: Piercing of the aortic valve leaflet

**Figure 3:**
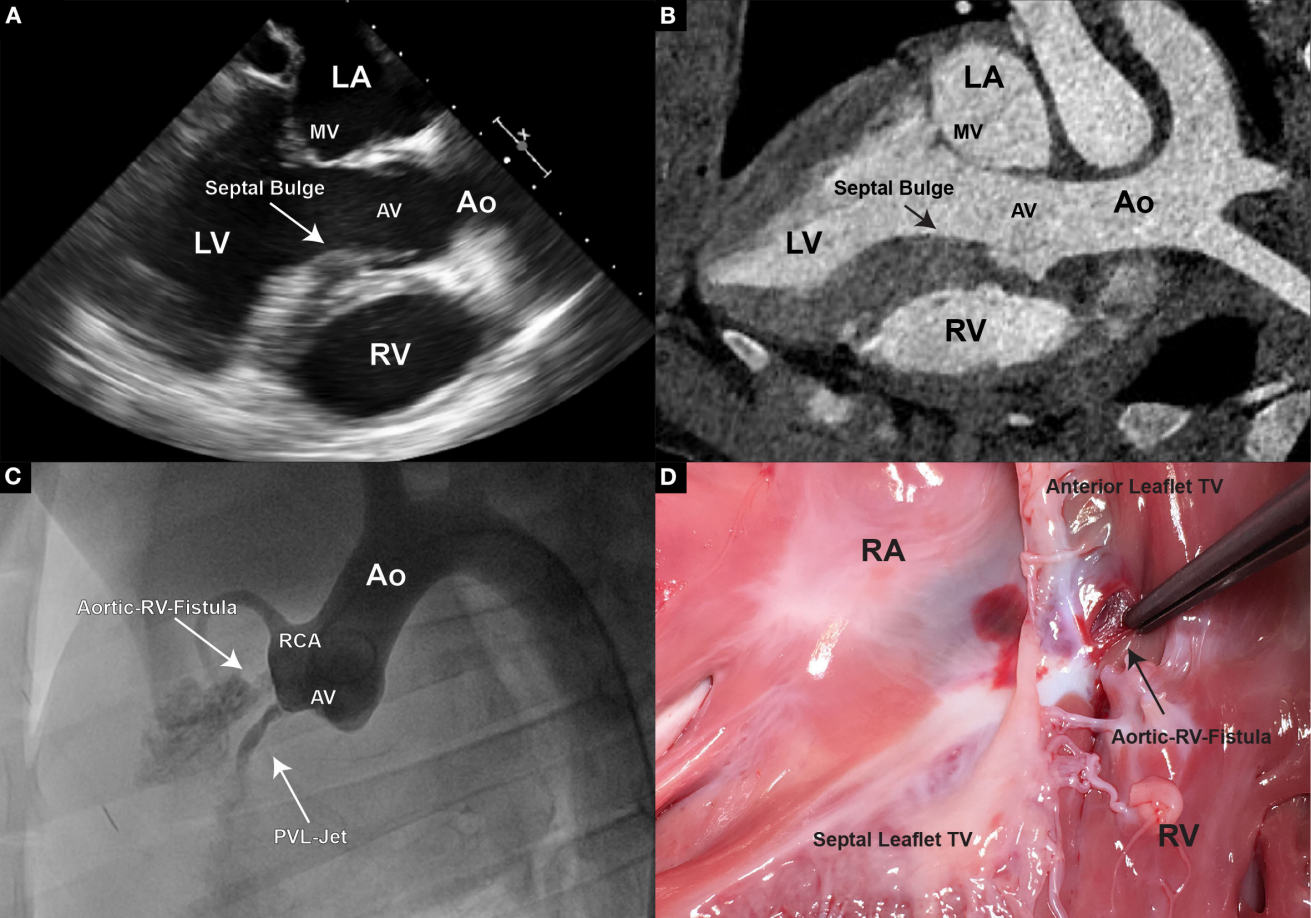
**(A)** In TEE, pigs display a unique feature of thickened septal wall. Especially the portion directly beneath the aortic valve, corresponding to the location of the RCC. Thickening of this region becomes prominent, especially during end-systole and early diastole; **(B)** In CT however, usually only general thickening of the septal wall can be observed, and often no prominent “bulge” is detectable; **(C)** An aortogram is performed following an erroneous piercing in the RCC region. A direct flow of contrast to the right ventricle could be observed demonstrating an aorto-right ventricular communication; **(D)** Upon pathological examination a fistula connecting the aortic root and the right ventricle was found.

After targeting the NCC or RCC, respectively, under the guidance of transesophageal and intracardiac echocardiography as well as fluoroscopy, the leaflet hinge was pierced with a stiff-end of a coronary guide wire (IRON MAN Guide Wire, 0.014″ 190 cm, Abbott Vascular, Santa Clara CA, USA) (Figures 2C,D). To improve the visibility of the guide wire in the echo image, the wire can be roughened with sandpaper. An apparent septal bulge or a septal muscular shelf can be found in pigs just beneath the RCC and is easily pierced accidentally (Figures 3A,B). Piercing the septal bulge will direct the guide wire toward the right ventricle and if unnoticed the created defect will cause an aorto-right-ventricular fistula (Figures 3C,D). After verifying the guide wire position in the left ventricle, the leaflet puncture site was then dilated with a 5-mm PTCA balloon (NC Emerge MONORAIL™ PTCA Dilation Balloon 5 × 12 mm, Boston Scientific Corporation, Marlborough, MA, USA). The balloon should be flossed multiple times in its inflated state through the leaflet to ensure sufficient defect size.

### Verification of PVL

**Figure 4:**
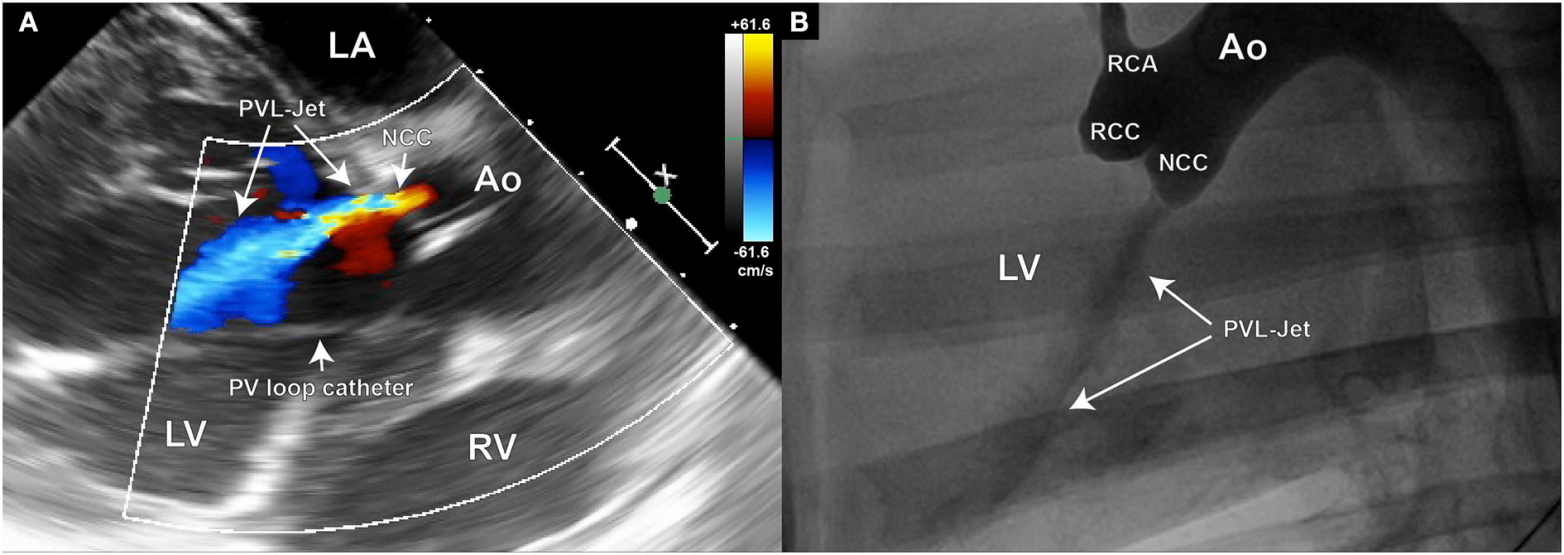
Echocardiography and aortography can be used for verification of the aortic valve defect; **(A)** Color Doppler image of the aortic insufficiency jet created in the NCC region of the aortic valve; **(B)** Aortographic image of the same animal in latero-lateral position also demonstrating the existence of the regurgitant jet. Such images can later be used for densitometric quantification of the regurgitant volume.

Doppler echography was used for the initial assessment of PVL presence and severity (Figure 4A). The width of the jet is thereby measured in relation to the left ventricular outflow tract (LVOT) diameter. A jet width of minimally 25% to maximally 65% of the LVOT is aimed for, as this is considered mild-to-moderate aortic regurgitation by the ASE guidelines on Aortic Regurgitation (29). An aortography can additionally be performed for periprocedural densitometric quantification of PVL (Figure 4B). The final quantification of PVL was done by post-processing particle tracking analysis on 4D Flow MRI datasets.

### Challenging LV performance

Aortic valve regurgitation causes an increase in end-diastolic pressure and volume in the left ventricle and an increase in the afterload (30). Dobutamine is a pharmacologic substance alternatively used instead of vasodilators, to induce cardiac stress (31). Dobutamine has a positive inotropic effect leading to an increase in cardiac output by selectively augmenting stroke volume and by a reflex decrease in total peripheral vascular resistance (32). Dobutamine is used clinically as a racemic mixture, whereby one of the stereoisomers has a strong agonistic adrenergic effect on α1 and a weaker effect on β1 and β2 activity, the other stereoisomer predominantly stimulates β1 and β2 adrenoreceptors and exhibits α1 antagonist activity (33). Thus, causing a null α1-mediated effect on the vasculature, a β2-induced increase in heart rate, and a positive inotropic effect mediated by all three receptors (33).

In this study, a dobutamine stress test was performed at baseline, after induction of PVL, and during MRI flow measurements. Dobutamine (Dobutrex, Teva Pharma AG, Basel, Switzerland; 0.5 mg/ml) was administered over a continuous pump infusion intravenously to effect. A 30% increase in heart rate was targeted and maintained over 10 min (PV loop) or for the duration of the MRI flow acquisition.

Phenylephrine is a direct-acting sympathomimetic amine that functions as an α1-adrenergic agonist. The described α1-adrenergic effects are thought to cause venoconstriction leading to a temporarily increased preload and to a larger extent arterial constriction which will increase systemic vascular resistance and afterload (34). In pigs, the administration of phenylephrine is associated with an increase in systemic arterial blood pressure, stroke volume, and cardiac output with no changes in heart rate. A preload-enhancing effect was observed in low-dose phenylephrine administration while higher doses appeared to increase contractility in a load-independent manner (35). In the presented study, a phenylephrine challenge was also performed at baseline, after induction of PVL, and during MRI flow measurements. Phenylephrine (Neo-Synephrine HCl, Ospedialia AG, Hüneberg, Switzerland, 0.1 mg/ml) was also administered over a continuous pump infusion intravenously to effect. This time a 30% increase in mean arterial pressure was targeted and maintained for over 10 min (PV loop) or the duration of the MRI flow acquisition, respectively.

### Magnetic resonance imaging

Due to animal size and weight, MR imaging was performed on a clinical 3T system. The animals were placed in the right lateral position. During the measurements, the animals should be mechanically ventilated and blood pressure as well as endtidal CO_2_ (etCO_2_) should be monitored. Ventilator and monitoring equipment is commonly placed outside the Faraday's cage, therefore, etCO_2_ monitoring is performed *via* a 14-m Heidelberger line and for mechanical ventilation, approximately 2 m × 12 m hoses are needed.

All imaging is commonly performed during ventilated breathing and cardiac synchronization by a pulse oximetry unit clipped to the animal's tail.

Functional imaging consisted of clinically used balanced steady-state free precession imaging in two-, three-, and four-chamber views as well as in short axis view covering the entire left ventricle. Imaging parameters were as follows: Field of view: 300 × 300 × 96 mm, spatial resolution 2 × 2 mm^2^, slice thickness 8 mm, TR/TE 2.7 ms/1.35 ms, retrospective triggering, temporal resolution 31 ms, and under sampling factor 2.

The 4D flow imaging is performed using a sparsely sampled pseudo-spiral Cartesian 3D sampling pattern using golden angle increments (36, 37). The frequency encoding direction is aligned to the left-right direction in order to capture chest wall motion and the imaging volume is planned parallel to the four-chamber view. Imaging parameters were as follows: FOV 300 × 410 × 90 mm, spatial resolution 2.5 × 2.5 × 2.5 mm, excitations per shot 5, acquired temporal resolution 21 ms, TR/TE 3.9/2.2 ms−4.2/2.5 ms. To estimate the maximal velocity expected and adjust the velocity encoding strength (venc) a quick 2D through-plane velocity encoded scan was performed at three slice positions: distal, within, and proximal of the aortic valve. The applied venc's for 4D flow imaging in our setting were: 140–180 cm/s (baseline), 150–180 cm/s (Phenylephrine), and 180–280 cm/s (Dobutamine).

Local low-rank image reconstruction can be performed offline within the MRecon framework (GyroTools LLC, Zurich, Switzerland) incorporating the Berkeley Advanced Reconstruction Toolbox (BART) (36, 37). Two breathing motion states should be reconstructed to minimize breathing artifacts with an under-sampling factor of approximately six each.

### Euthanasia and gross pathology

**Figure 5:**
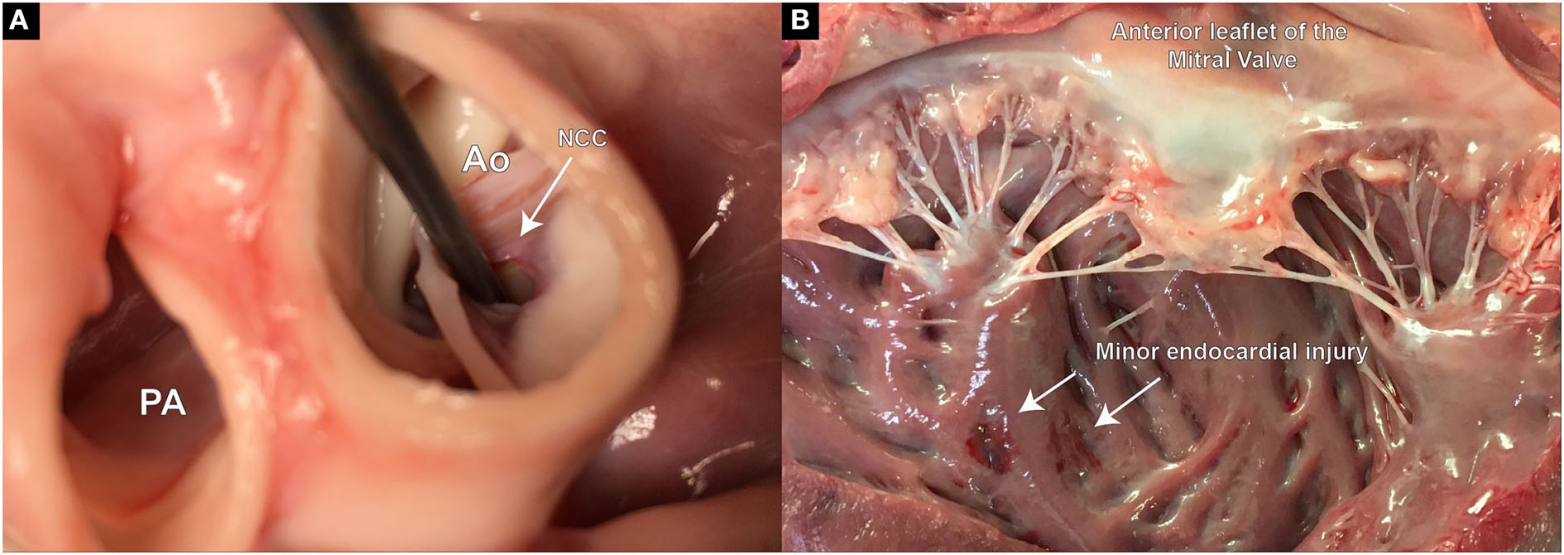
Postmortem examination of defect size, location, and potential extra-valvular injuries created during the procedure. **(A)** Demonstrates the defect precisely placed at the hinge point of the aortic leaflet in the RCC region; **(B)** Slight injury on the endocardial surface of the left ventricle, most likely caused during the insertion of the piercing guide wire.

At the end of the study, pigs were fully heparinized (300 IU/kg) and euthanized under general anesthesia by administering an overdose of sodium pentobarbital (Eskonarkon^®^ad.us.vet., Streuli Pharma AG, Uznach, Switzerland;75 mg/kg body weight) intravenously. The heart was fully excised through a sternotomy and the aortic arch opened longitudinally. Leaflet defect size, location, and further injury to the LVOT or endocardial tissue are optically assessed and documented (Figures 5A,B).

## (Anticipated) results

### Creation of precisely placed aortic PVL defects with defined size is possible

**Figure 6:**
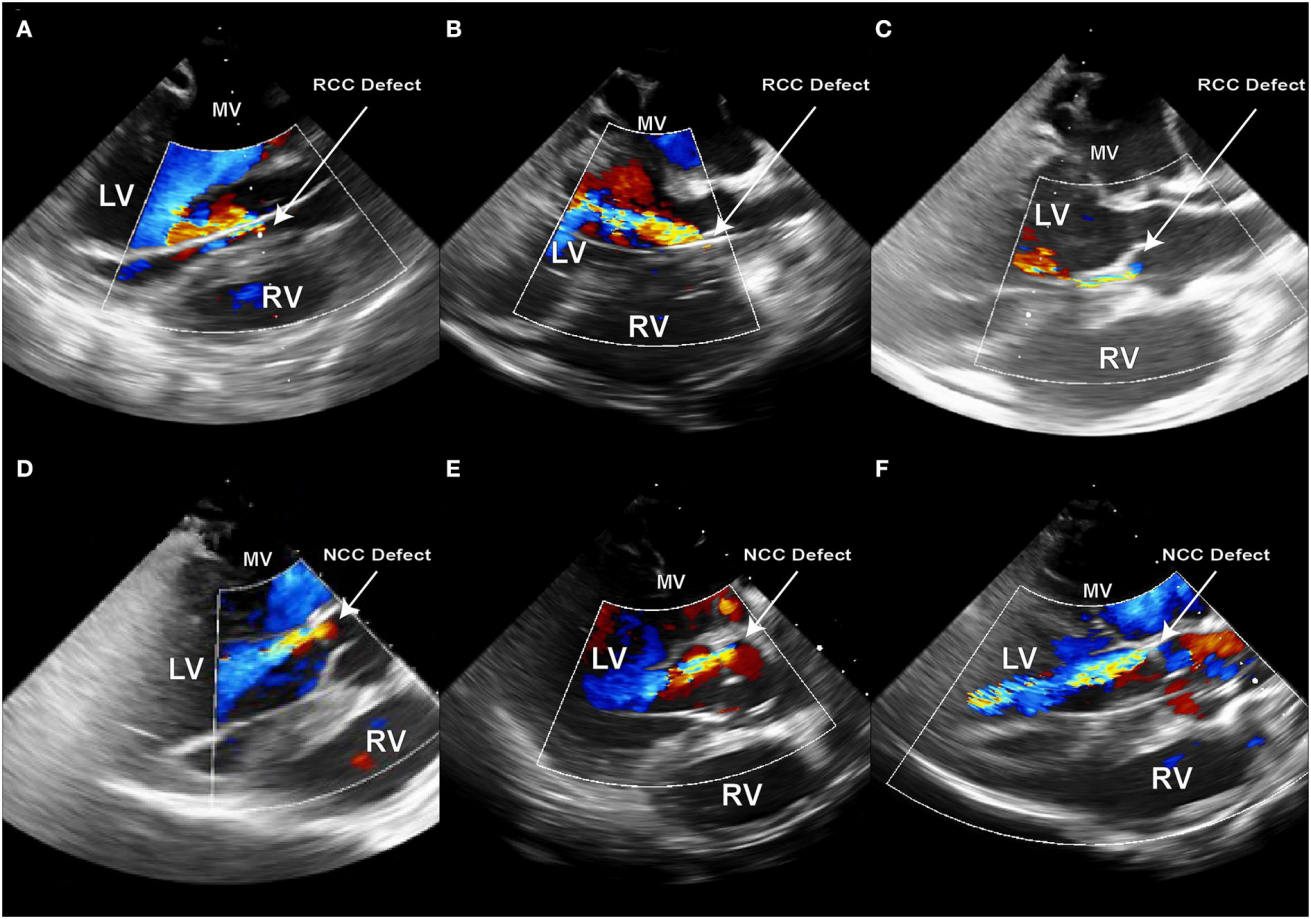
Echocardiography immediately after the creation of the PVL defect in sx consecutive animals. **(A–C)** Defects created in the RCC annular region; **(D–F)** Defects created in NCC annular region; PVL jet originating from the NCC region have a trajectory along the anterior leaflet of the mitral valve and can be fully visualized along their path in the LV. RCC jets on the other hand either flow along the septum or at a steep angle across the LV.

To model the PVL of the aortic valve, the defect needs to be placed at the hinge point between the leaflet and the valvular annulus. Care must be taken for the defects not to be placed within the leaflet tissue, as this would not mimic the clinical situation. In our study, the defect-target areas were set at the hinge points of either the right coronary cusp (RCC) or the non-coronary cusp (NCC) of the aortic valve, respectively, as they represent the major predilection sites for PVLs in the clinic. In the reported six animals, three NCC and three RCC defects were targeted and successfully created using the described method. It is worth noting that intraprocedural imaging and procedural guidance were more challenging for the RCC defects, leading to longer procedural duration in this group. Immediately following the defect creation, resulting aortic regurgitant jets could be observed in TEE in all six animals (Figures 6A–F). However, due to the anatomical position of jet origin and their trajectory in the LV, NCC regurgitant jets could be fully visualized whereas it was only partially possible for the RCC jets (Figures 6A–C). Moreover, the postmortem analysis showed that 3/3 NCC and 2/3 RCC defects were precisely placed within the narrow hinge region in the middle of the valve leaflet. Due to an erroneous puncture in 1/3 RCC that created an aorto-right ventricular fistula (Figures 3C,D) the RCC target location was slightly moved laterally and the defect was placed 5 mm lateral from the middle point. Yet, all animals remained hemodynamically stable after defect creation, and during the subsequent measurements.

### Stable positioning of the PV loop catheter enables measurements of the acute effects of PVL jets on ventricular work and direct comparison to baseline state

**Figure 7:**
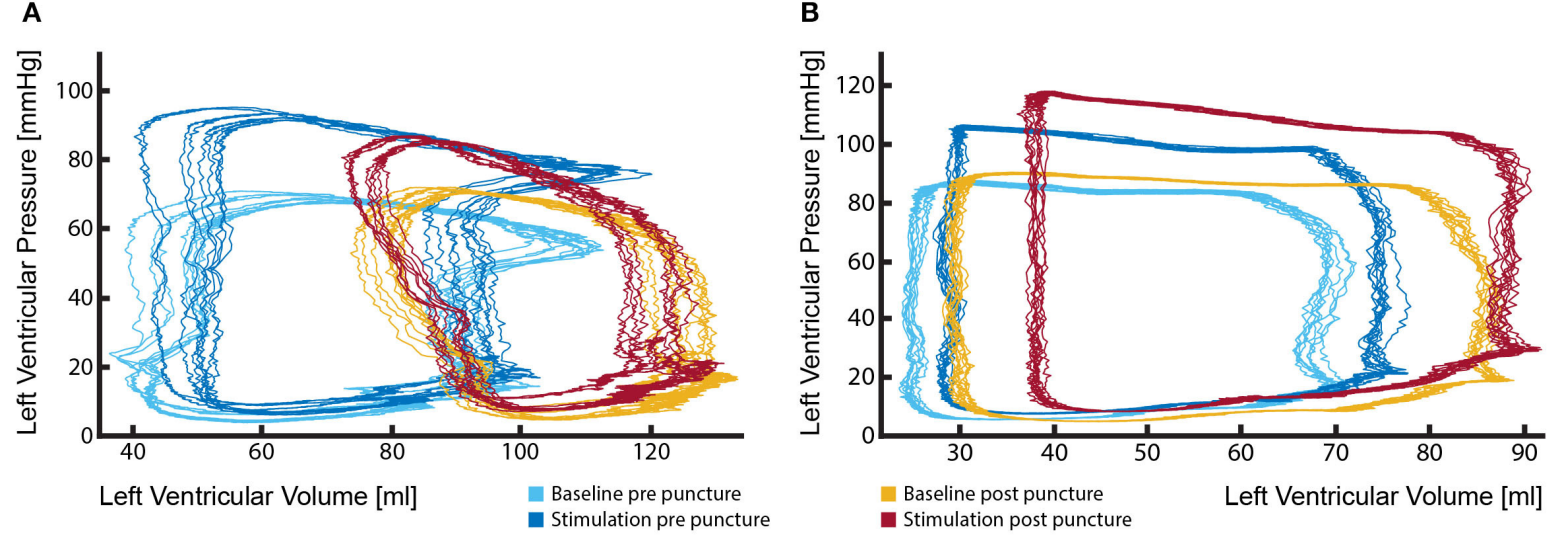
Stable position of the PV loop catheter during the entire procedure is fundamental for the evaluation of the effects of aortic regurgitant jets on left ventricular performance. **(A)** Marked change in PV loop curves following the removal and reinsertion of the catheter after the defect creation. Such changes render the comparison of the LV performance between the healthy state (light and dark blue loops) and aortic insufficiency state (red and yellow loops) particularly challenging and potentially misleading; **(B)** By keeping the catheter in a stable position during the entire procedure reliable measurements could be performed, delineating effects that aortic regurgitation causes to LV performance (light blue vs. yellow loops). Furthermore, investigations under pharmacologically changed hemodynamic conditions are possible (dark blue vs. red loops).

The fact that severe PVL and/or aortic insufficiency has a negative effect on patient survival and quality of life has been widely reported. However, the impact of mild-to-moderate jets is less clear and patients display a wide range of symptoms. It is important to understand what impact these jets might have on left ventricular work parameters. However, this is only possible if the PV loop catheter is able to record closely comparable signals before and after the defect creation and if the hemodynamic parameters (such as arterial blood pressure, heart rate, and cardiac contractility) remain within very narrow margins. PV loop catheter removal during the defect creation procedure would make this comparison almost impossible (Figure 7A). To avoid catheter removal, our initial approach was the transseptal placement of the PV loop catheter in the LV. However, signals obtained by such an approach were of insufficient quality and the catheter could not be held in a stable position, leading to strongly variable output. Consequently, we developed a method of placing the PV loop catheter in the aortic valve commissure between LCC and RCC. In this position, the catheter remained stable even during the transcatheter PVL creation. Hence, a direct comparison between the healthy (baseline) and PVL state could be made (Figure 7B). More importantly, PV loop catheter position and the signal remained stable during the whole study, even throughout the drug-induced hemodynamic changes (Figure 7B).

### Drug-induced changes in afterload and heart rate enable the study of the aortic PVL under clinically relevant conditions

Increased afterload markedly contributes to the severity of PVL as it aggravates aortic valve regurgitation. In an experimental setting, phenylephrine, as an α1-adrenergic agonist, can be used to induce peripheral arterial constriction, which will increase systemic vascular resistance and afterload. The direct effect of increased vascular resistance on regurgitation volume can be assessed and correlated.

An increased heart rate in patients with aortic valve regurgitation has previously been found to decrease regurgitant flow per stroke and per unit time (38) and a reduction in left ventricular end-diastolic pressure (LVEDP) (39). In a previous study, dobutamine in healthy pigs has shown to increase heart rate and ejection fraction, with a decrease in left ventricular end-diastolic volume and end-systolic volume, and no change in stroke volume (40). Dobutamine has furthermore shown to change the intra-cardiac blood flow pattern in healthy pigs (40).

The effect of dobutamine seemingly counteracting aortic valve regurgitation-induced increase in LVEDP and afterload on cardiovascular physiology and intra-cardiac blood flow pattern. It thereby examines important aspects of the clinical situation in patients, in which aortic valve regurgitation may manifest in an increasing afterload mitigating arterial hypertension or an increased heart rate induced by new onset tachycardiac atrial fibrillation. The latter is present in around 5% of transcatheter valve implantation and in a remarkable 40% of surgical aortic valve replacements (1). Both clinical conditions are proven cardiovascular risk factors by themselves and may potentially have a negative synergistic effect on the increased mortality and morbidity induced by PVL.

### Echocardiography and densitometry provide a reliable solution for experimental assessment of PVL severity in a porcine model

Although echocardiography is challenging in the porcine model, with moderate technique adaptations most of the interventional-relevant cardiac structures can be readily visualized (23). However, providing echocardiographic windows appropriate for Doppler flow assessments is much more challenging. Moreover, PVL jets have a distinct trajectory within the 3D space of the LV. Due to a limited echo–window in TEE, it is often impossible to follow the jet in its full length. In our study, this was particularly challenging in the RCC group, where already within the LVOT the jet would exit the echo plane (Figures 6A–C). However, for severity assessment of aortic PVL the width of the jet within the LVOT is often used as a reliable parameter in the clinic (41, 42). Such measurements are reproducibly achievable by TEE in pigs and could demonstrate that 6/6 defects created have caused mild-to-moderate aortic regurgitation. On the other hand, densitometry is much less sensitive, albeit not completely insensitive, to projection angles and imaging windows. In contrast to humans, the best projection for densitometric imaging of aortic insufficiency is a latero-lateral projection. Care has to be taken that no overlap of cardiac and aortic structures is imaged and the scapulae are not in the frame. Furthermore, the high volume (20 ml in 1.5 s) contrast agent injection in the aortic root required for the densitometric analysis could lead to a short increase in the local aortic pressure. As the difference in aortic and LV diastolic pressures is the driving force behind the aortic regurgitation, such pressure increase on the aortic side exactly at the time point of evaluation could potentially lead to PVL overestimation. Although a clear correlation between the echocardiographic and densitometric regurgitant volume assessment has been shown in patients (43), there are currently no data from animal trials.

### The 4D flow MRI delivers answers to correlate disturbed ventricular blood flow patterns and potential adverse remodeling

Diastolic intraventricular blood flow acquired with 4D MRI has shown to be altered in pigs with mild-to-moderate PVL in a defect site (NCC vs. RCC) dependent manner (3) as well as under dobutamine stress alone when compared to healthy pigs (40). Deviations from physiologic diastolic blood flow are described to result in an increased energy dissipation within the LV (44). The effect of pharmacologically increased afterload or heart rate on intra-cardiac flow patterns in 4D MRI can give further insights into kinetic energy changes within the left ventricular blood pool in mild-to-moderate PVL.

## Discussion

Paravalvular regurgitation or leak (PVL) is a complication associated with transcatheter valve replacements and causes turbulent blood flow below the respective valve with varied clinical consequences (42). Porcine models for the study of the effects of altered blood flow and hemodynamics on cardiac health after induction of PVL are of great interest. However, hemodynamic studies are easily biased by insufficient considerations of confounding factors. Aside from selecting pigs of appropriate size and weight, the health status can have a significant impact on cardiovascular and hemodynamic properties and should be assessed prior to animal purchase. On commercial farms, pigs are often subjected to handling practices causing acute stress (45). The major pathways activated by stressors are the hypothalamic–pituitary–adrenal (HPA) axis and the sympathetic nervous system (46). Stress-free handling of pigs used for hemodynamic studies is therefore essential. An appropriate acclimatization time for the animals at a new facility should be granted as during transportation and after stabling, pigs are confronted with a variety of stressors leading to stress-induced changes in the cardiovascular, endocrine, immune, and reproductive systems (47–49). Transporting and housing pigs in familiar groups can attenuate social stress (50). To prevent heat or cold stress in pigs, it is critical to base housing temperature on the energy content of the diet, the pig's growth status, and group housing (51). Utilizing stressed animals before their physiological status normalizes can have considerable and unintended effects on cardiovascular research results (49). While primary mediators of stress (e.g., catecholamines and glucocorticoids) will return to physiological values within 24 h, changes in the immune and endocrine systems may take up to 7 days to normalize (49). The diet changes should be induced gradually during the time of acclimatization. Animals displaying diarrhea should be excluded from any study as diarrhea contributes to an increased loss of electrolytes and water over the bowel lumen (52) causing a disruption in homeostasis and acid-base balance. These changes have been shown to affect cardiac contractility and rhythm with a potentially fatal outcome (53, 54). Studies in rats showed osmotic diarrhea, often accompanied by a reduction in food intake, to cause a reduction in heart weight and protein content by reduced protein synthesis (55). Various anesthetic protocols are used in laboratory pigs, each having its advantages and disadvantages. The requirements for the anesthetic protocol are particularly high when conducting hemodynamic studies. Adequate intramuscular sedation is commonly required in pigs, for safe handling and to place an intravenous catheter in the auricular vein for induction of general anesthesia. Dissociative anesthetic drugs like ketamine and tiletamine are thereby often combined with sedative agents like azaperone, midazolam, zolazepam or α-2 agonists, such as detomidine, medetomidine or dexmedetomidine (56–59). As α-2 agonists are described to induce adverse cardiovascular effects such as bradycardia, increased systemic vascular resistance, reduced cardiac output and oxygen delivery (60, 61), they are less suitable for cardiovascular research. Anesthesia can be maintained with inhalation agents or intravenous drugs (TIVA), or a combination of both. Isoflurane and other volatile anesthetics have dose-dependent effects on the hemodynamic and respiratory function which can be attenuated when combined with partially intravenous anesthesia and analgesia (PIVA) (62, 63). Anecdotally, the ventricular fibrillation (VF) threshold in pigs is markedly lower than in humans. One plausible reason is the Purkinje system, which is located transmurally in pigs and subendocardial in humans (64). With pigs being more prone to VF and other cardiac arrhythmias during cardiac interventions, antiarrhythmic agents are an integral part of a successful anesthetic protocol. Amiodarone is a potent antiarrhythmic drug successfully used in the treatment of ventricular and supraventricular arrhythmias in pigs (65, 66). However, a study in dogs showed a dose-related decrease in both coronary and systemic vascular resistance, as well as a decrease in cardiac contractility after the administration of amiodarone (67). Hemodynamic side effects resulting in a decreased aortic (systolic, diastolic, and mean) and left ventricular (systolic and end-diastolic) pressure are also described in humans (68). The hemodynamic effect of amiodarone as a potent antiarrhythmic drug in pigs should therefore be taken into consideration. Porcine models of aortic PVL are commonly created by the implantation of a prosthetic valve (TAVI) (4). Prosthetic aortic valves can thereby be implanted either in an antegrade matter trans-apically through a subxiphoid access or mini-thoracotomy in the 6th intercostal space (69) or retrograde through a percutaneous access sheath in the femoral artery (70). Percutaneous approaches are often hampered by the need for large access sheaths increasing the risk for arterial dissection and perforation (71). Transapical approaches on the other hand require the placement of a large access sheath through the left ventricular apex. The puncture site is closed by a purse-string suture after successful valve implantation, often causing apical regional wall motion abnormalities with an overall decrease in left ventricular function due to myocardial injury (72). Porcine TAVI-induced PVL models are therefore impractical for hemodynamic studies. Furthermore, although there is no risk for prosthetic valve movement or dislodgement using an MR system operating at 1.5 T or less, the presence of an artifact may affect the diagnostic imaging quality and 4D flow assessment through the prosthetic valve (73). The described minimally invasive and reproducible porcine model is therefore ideal for a detailed investigation of hemodynamic effects and the short-term impact caused by mild-to-moderate PVL in patients. Despite a high success rate (6/6) in creating mild-to-moderate aortic regurgitation, specifically targeting leaflet hinge points, especially in the RCC region remains somewhat challenging and not completely devoid of complications. Where the mid-points of the NCC leaflet hinge are clearly visible and easily reachable with the described method and equipment, setting of RCC defects requires adaptations in intra-procedural imaging as well as interventional technique. Moreover, the unique porcine feature of the muscular septal shelf (septal bulge) positioned just beneath the RCC aortic leaflet could be the cause of complications if erroneously pierced (21). In our study, this occurred in one-third RCC targeted defects. Furthermore, effects of such a structure on the trajectory and path of the PVL jet originating from RCC can currently not be excluded and have to be considered as a limitation to the translatability of the results. Yet, due to the minimally invasive nature of the PVL defect creation technique (the procedure requires one femoral vein and two arterial accesses) the model described here potentially offers a viable platform for long-term investigation of cardiac remodeling and other adverse effects caused by aortic PVL. In accordance to the 3R principles, such model would represent a substantial refinement in comparison to the surgical or even transcatheter implantation of a defective valve (4). Moreover, post-operative manipulations necessary for the administration of anticoagulation therapy following valve implantations are not required in our model as there is no thrombogenic foreign material. As the PVL orifices represent areas of high-velocity flow, it is not expected that they would spontaneously heal in our model. However, such aspects as well as the induction and progression of adverse cardiac remodeling need to be carefully evaluated in studies specifically designed for that purpose. Pressure–volume loops (PV loops) are the gold standard for real-time load-dependent and load-independent measures of the left ventricular systolic and diastolic function. A practical guide on PV loop measurements in pigs has recently been published (27). However, PV loop measurements are easily disturbed through further interventions, such as aortic valve leaflet piercing. To avoid interference at the aortic valve and to avoid moving the PV loop catheter between measurements, transseptal placement of the PVL catheter was considered. Transseptal puncture was performed under ICE guidance and verified by left atriography. However, with the left atrial height being much lower in pigs than in humans (74) and the porcine fossa ovalis being both deeper and more superiorly positioned, trans-septal placement of the PV loop catheter leads to a lateral displacement of the posterior leaflet of the mitral valve, causing the catheter to extensively move with the opening and closing of the mitral valve.

Further consideration when conducting hemodynamic studies in pigs has to be given to an optimized intra-operative fluid management, which is essential to maintain intravascular volume and hence cardiac preload and cardiac output. Longlasting anesthetic procedures tend to cause hypovolemia due to ongoing losses through mechanical ventilation and urinary output. Additionally, blood loss through the access sheath and catheters, which is further aggravated due to anticoagulation therapy, has to be accounted for. Intravascular volume status should be assessed and compensated throughout the study by combining laboratory parameters, fluid administration, and urine output measurements with monitoring of central venous pressure and cardiac output (75).

Despite taking into consideration all the described dos and don'ts in establishing a porcine model for PVL, substantial inter-individual variability in hemodynamic compensation to PVL in pigs is to be expected. It is all the more important to have a standardized and reproducible model of PVL to exclude procedural bias.

## Data availability statement

The original contributions presented in the study are included in the article/supplementary material, further inquiries can be directed to the corresponding author.

## Ethics statement

The animal study was reviewed and approved by Cantonal Veterinary Office Zurich, Switzerland (ZH213/2019).

## Author contributions

NC: conceptualization, study design, development of the animal model, surgical procedure, animal handling, preparation, and review of the manuscript. MW, LG, and MKo: development of animal model, surgical procedure, and preparation of the manuscript. NT, MH, AL, and TS: animal handling and anesthesia. MKu: study design and echocardiographic imaging analysis. CS: 4D MRI protocol and imaging procedure and preparation of the manuscript. VF and ME: data interpretation and critical review of the manuscript. All authors contributed to the article and approved the submitted version.

## Funding

This work was supported by a research grant from the Swiss Heart Foundation and was performed within the framework of the ETHeart initiative. Open access funding provided by ETH Zurich.

## Conflict of interest

The authors declare that the research was conducted in the absence of any commercial or financial relationships that could be construed as a potential conflict of interest.

## Publisher's note

All claims expressed in this article are solely those of the authors and do not necessarily represent those of their affiliated organizations, or those of the publisher, the editors and the reviewers. Any product that may be evaluated in this article, or claim that may be made by its manufacturer, is not guaranteed or endorsed by the publisher.
